# Recent advancements in the use of plastics as a carbon source for carbon nanotubes synthesis - A review

**DOI:** 10.1016/j.heliyon.2024.e24679

**Published:** 2024-01-13

**Authors:** Helen U. Modekwe, Michael O. Daramola, Messai A. Mamo, Kapil Moothi

**Affiliations:** a Renewable Energy and Biomass Research Group, Department of Chemical Engineering, Faculty of Engineering & the Built Environment, University of Johannesburg, Doornfontein Campus, 2028, Johannesburg, South Africa; b Department of Chemical Engineering, Faculty of Engineering, Built Environment and Information Technology, University of Pretoria, Private bag X20 Hatfield, 0028, Pretoria, South Africa; c Research Centre for Synthesis and Catalysis, Department of Chemical Science, Faculty of Science, University of Johannesburg, Doornfontein Campus, 2028, Johannesburg, South Africa; d School of Chemical and Minerals Engineering, Faculty of Engineering, North-West University, Potchefstroom 2520, South Africa; e Department of Chemical Engineering, Faculty of Engineering and the Built Environment, University of Johannesburg, Doornfontein campus, 2028, Johannesburg, South Africa

**Keywords:** Plastic-derived carbon nanotubes, Waste plastics, Synthesis conditions, Carbon nanotubes quality, Carbon nanotubes yield

## Abstract

Plastics, which majorly consist of polypropylene (PP), polyethylene (linear low-density polyethylene (LLDPE), low-density polyethylene (LDPE) and high-density polyethylene (HDPE)), polystyrene (PS), polyvinyl chloride (PVC), polyethylene terephthalate (PET), etc., are the most abundant municipal solid wastes (MSW). They have been utilized as a cheap carbon feedstock in the synthesis of carbon nanotubes (CNTs) because of their high hydrocarbon content, mainly carbon and hydrogen, especially for the polyolefins. In this review, the detailed progress made so far in the use of plastics (both waste and virgin) as cheap carbon feedstock in the synthesis of CNTs (only) over the years is studied. The primary aim of this work is to provide an expansive landscape made so far, especially in the areas of catalysts, catalyst supports, and the methods employed in their preparations and other operational growth conditions, as well as already explored applications of plastic-derived CNTs. This is to enable researchers to easily access, understand, and summarise previous works done in this area, forging ahead towards improving the yield and quality of plastic-derived CNTs, which could extend their market and use in other purity-sensitive applications.

## Abbreviations

AAO: Anodic aluminium oxide
CB: Carbon black
CNFs: Carbon nanofibers
CNMs: Carbon nanomaterials
CNOs: Carbon nano-onions
CNS: Carbon nanosphere
CNTs: Carbon nanotubes
CS-CNTs: Cup-stacked carbon nanotubes
CVD: Chemical vapour deposition
CVD-FBR: Chemical vapour deposition-fluidized bed reactor
DWCNTs: Double-walled carbon nanotubes
ER: Equivalent ratio
EVA: Ethylene-vinyl acetate
GNSs: Graphene nanosheets
GPPS: General-purpose polystyrene
HDPE: High-density polyethylene
HER: Hydrogen evolution reaction
HIPS: High impact polystyrene
LDPE: Low-density polyethylene
LLDPE: Linear low-density polyethylene
MB: Methylene blue
MP: Mixed plastics
MSI: Metal-support interaction
MWCNTs: Multiwalled carbon nanotubes
NAAM: Nanoporous anodic alumina membranes
ORR: Oxygen reduction reaction
PCNS: Porous carbon nanosheets
PE: Polyethylene
PET: Polyethylene terephthalate
PP: Polypropylene
PP-MA: Polypropylene maleic anhydride composite
PSW: Plastic solid wastes
PU: Polyurethane
PVC: Polyvinyl chloride
PVP: Polyvinylpyrrolidone
SWCNTs: Single-walled carbon nanotubes
SCCM: Standard cubic centimetres per minute

## 1 Introduction

The emergence of plastic has contributed immensely to modern society and the standard of living. Their application in almost all major daily and commercial activities in packaging, automobiles, construction, and appliances cannot be overemphasised due to their lightweight and portability, durability, relatively low-cost, corrosion and chemical resistance, etc. These ample qualities and properties reflect their extremely geometric increase in plastic production. For example, polyolefins such as polypropylene (PP) and polyethylene (PE) are the most prevalent synthetic polymers with the highest world production capacity of about 63 %, with PP dominating over 56 metric megatons and PE 76 metric megatons of the world plastic market [1,2]. The increase in production is reciprocated by a corresponding incessant increase in plastic waste globally.

It has been established that the highest-produced plastic materials also constitute the highest waste; hence, municipality plastic solid wastes (PSW) constitute packaging wastes emanating from PP, PET, and PE, which contribute to about 65 % of total plastic solid wastes [3]. Globally, around 42 % of all plastic ever made is used in packaging [[3], [4], [5]]. Most of these polymers are recyclable and for single use; therefore, they have a short-useful life span, usually less than one year, which is one of the reasons they are problematic and are everywhere, causing environmental pollution. Globally, plastic pollution is a great concern considering their non-biodegradability over a long time, leading to their stockpiling in landfills and being a major source of plastics in the marine environment, where they cause severe environmental and health problems for marine animals and also deteriorate the waterbodies [6]. Between 1990 and 2017, about 172 million tonnes of plastics (comprising both primary polymers and plastic products) were imported into the African continent from the United Kingdom, Germany, the United States, etc., while about 15 million tonnes of plastics were also produced within Africa [7]. It is estimated that plastics imported into South Africa account for about 13.7 million tonnes, of which over 6.3 million tonnes of waste plastics generated are been mismanaged yearly, with about 0.9–2.5 million tonnes of these plastic wastes accumulating in the waterbodies [7].

### 1.1 Waste plastics disposal and treatment routes

Landfilling is the most practiced and relatively cheap disposal method all over the world, with 79 % of all plastics being accumulated in landfills globally [8]. However, landfilling takes up more and more land, leading to various environmental problems ranging from contamination of the environment to additive leaching due to their chemical nature [9], emission of greenhouse gases (e.g. carbon dioxide), and other health-related problems such as respiration risks etc. Also, during run-offs and storms, these materials find their way into water bodies, causing serious health risks arising from the ingestion of plastics by aquatic and terrestrial animals [10,11].

One of the proposed routes proven to be permanent avenues of getting rid of PSW from our environment is recycling, which could be by mechanical, chemical (feedstock), or thermal (energy recovery or incineration) routes [12,13]. In mechanical recycling, waste materials are converted into new consumer products with the addition of pigments (dyed) and additives by mechanical methods [14]. South Africa, for instance, recorded great progress in mechanical recycling rates for packaging waste (LDPE) in 2018, with more than 334,000 tons of the waste recovered back to their raw materials, giving South Africa a recycling rate of about 46.3 % [15,16] compared to Europe, which sits at approximately 31.1 % [15]. Nevertheless, about 70 % of South Africa's waste is still being recovered from landfills, making this practice costly [15]. Regardless of this commendable recycling rate, creating a market for these recyclates (secondary products) is challenging because pigmented and dyed plastics have low market values as recycled products lose some of their strength and firmness. Additionally, recycling is costly and is limited to a certain number of cycles; hence, manufacturers prefer transparent plastics to dyed and pigmented ones [1,17]. However, mechanical recycling is a temporary means of postponing waste rather than eliminating waste disposal and accumulation; hence, it is not sustainable. Globally, about 9 % of all plastics have been recycled, with only around 10 % of them being recycled more than once [3,18]. Energy recovery or incineration has been utilized around the world owing to the high energy content of plastics, although issues have been raised about the cost and contribution to harmful emissions [19,20]. Chemical and thermal recycling (tertiary recycling) involves pyrolysis, gasification, and hydrothermal processes where monomers such as methane, propylene, ethylene, ethene, ethane, dienes, etc from which the plastic polymer was originally made are regenerated. Since these monomers are hydrocarbon-based, they are utilized as relatively cheap carbonaceous feedstocks for value-added carbon-based nanomaterials [21,22]. Chen et al. [20] reviewed the carbonization of plastic wastes as a method of reusing and converting wastes into valuable carbon materials. This idea will not only reduce plastic waste accumulation and environmental problems associated with its disposal but also create a new market for products made of recycled plastic and probably reduce the price of carbon nanomaterials (CNMs).

A great feat has been made so far over the years in transforming plastic materials into value-added CNMs with various morphologies and structures such as carbon-nanofibers (CNFs) [23,24], CNTs [[25], [26], [27]], cup-stacked CNTs (CS-CNTs) [28,29], carbon nanospheres (CNSs) [30], porous carbon nanosheets (PCNS) [31], graphene nanosheets (GNSs) [32,33], etc. Several reviewed works in the open literature have been carried out on synthesizing CNTs with other materials (hydrogen, and/or some carbon nanomaterials) from different types and mixtures of plastics, with much emphasis on catalysts and substrates, parametric conditions, methods of synthesis, reactor design and types, conversion processes, etc. [22,[34], [35], [36], [37], [38], [39]]. Similarly, a few studies have also synthesized heteroatom-doped CNTs from plastics [[40], [41], [42]]. Heteroatom doping has been studied as an effective way to modify the physicochemical properties of carbon-based nanomaterials for different applications. Dopants such as nitrogen, boron, phosphorus, and sulphur are introduced into the CNM lattice using heteroatom-containing precursors (such as melamine) through post-treatment synthesis or by in situ synthesis routes [42].

Herein, the authors emphasize the work done on the conversion of plastics into CNTs (only), with a more comprehensive focus on the catalysts and catalyst supports (their loading, and plastic/catalyst ratio), and the methods used in catalyst preparation because the selectivity, nature, and properties of synthesized CNTs (morphology and diameter) are heavily controlled by the catalyst characteristics such as the size of the active metal particle, etc. In addition, progress made so far in reactor designs, other parametric conditions, and practical applications of plastic-derived CNTs over the years in realizing and achieving waste-to-wealth, circularity, and environmental sustainability are highlighted. This idea has been harmonised and provided in detail with appropriate published data from recent literature.

### 1.2 Carbon nanotubes synthesis from waste plastics

Carbon nanotubes are a 1D (one dimensional) allotropic form of carbon with lengths and diameters existing in the order of micrometers and nanometers, respectively. They are essentially described as long hollow hexagonal structures (graphene) rolled up into cylindrical tubes [[43], [44], [45]]. Single-walled carbon nanotubes (SWCNTs), double-walled carbon nanotubes (DWCNTs), and multi-walled carbon nanotubes (MWCNTs) are the three major types of CNTs based on the number of walls they have. SWCNTs are single graphene sheets rolled into tubes with a diameter between 0.4 nm and 3 nm, while MWCNTs are piled layers of concentric graphene sheets rolled into cylindrical tubes with a diameter up to 100 nm [46,47]. These materials exhibit superb characteristics such as high surface area, high aspect ratio, good transport medium, chemical stability, good electrical conductivity, excellent mechanical strength, and stiffness, etc. [48]. These properties have also enabled them to find different applications in different fields, such as reinforcement material in composites, nanoelectronics, sensors, field emitters, tissue engineering, hydrogen and energy storage, catalyst supports, selective absorbents in wastewater treatment and carbon dioxide capture, etc. [46,48].

**Fig. 1:**
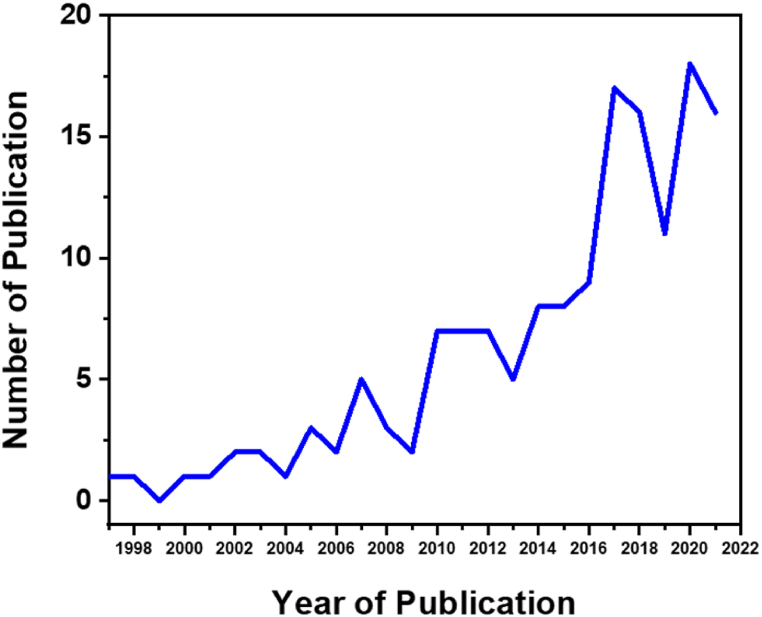
Number of publications (research articles) on carbon nanotubes synthesis using plastics as carbon source.

CNTs are grown in the presence of carbonaceous feedstock, heat, and a suitable catalyst [22]. The development of CNTs from plastics is extremely dependent on engineering highly active catalysts with extended catalyst life (which would be able to withstand the enormous carbon deposition), optimized experimental parameters, and the development of proper reactors. Fig. 1 reveals the relatively rising trends in the number of research articles published on the synthesis of carbon nanotubes (CNTs) from waste plastics between 1997 and 2021. Data utilized for the aforementioned period were obtained from Scopus, Google Scholar, and Web of Science scientific databases.

## 2 Role of catalysts

### 2.1 Effect of catalysts in CNTs synthesis

Catalyst design is very crucial in controlling CNTs growth. The stability, activity, and selectivity of catalysts are heavily influenced by their composition, pre-treatment conditions, preparation methods, interaction with support, and morphology [49]. A highly stable and active catalyst must be introduced to obtain high yield as well as good-quality CNTs. The overall efficiency of a catalyst for CNTs growth is measured by its ability to withstand a longer lifetime amid huge carbon production [50,51]. The characteristics of the catalyst directly affect the stability and activity of the catalyst, which also depend on the electronic state of the metal particles and the interaction between the metal and support catalysts. Other features and conditions that could influence catalyst activity and stability are crystallinity, crystalline size, dispersion of metal particles, textural properties and pore geometry, catalyst composition, catalyst preparation method, pre-treatment conditions (calcination and reduction temperatures, etc.) [50,52,53].

Transition metals (nickel, iron, and cobalt) are predominantly employed as catalysts for CNTs growth. The transition metal's ability to decompose carbon compounds results in the formation of carbides, and the ease with which carbon rapidly diffuses through the metal forms the basis for its catalytic activity [52]. The VLS (vapour-liquid-solid) mechanism is the commonly used model to explain the growth mechanism of CNTs [52,54]. In the VLS mechanism, the carbon-containing gaseous precursor is first absorbed and dissociated on the surface of the catalyst particle to form carbon atoms. Then, the formed carbon atoms dissolve in the bulk of the catalyst nanoparticles to form metastable carbide, which diffuses through the catalyst nanoparticle and precipitates at the surface of the nanoparticle, forming carbon nanofilaments [52,54]. However, the VLS growth mechanism has been controversial and challenged based on the driving force that propelled the carbon atoms to diffuse through the catalyst particle [54]. Hence, depending on the synthesis method and the characteristics of the catalyst, understanding the CNTs growth mechanism is crucial in tailoring CNTs properties, yield, and quality.

Blending two or more transition metals has been studied to be more efficient for CNTs production than one metal alone. Other metals other than nickel, iron, and cobalt have also been used as cocatalysts. These metals, such as molybdenum, magnesium, copper, etc., are generally mixed in different ratios to enhance the performance of the parent catalyst towards the production of better-quality CNTs and, at the same time, lower the synthesis temperature and process kinetics [13,52]. Even when these transition metal catalysts catalyse CNTs’ growth, they do not precisely yield similar results due to the electronic structure between different metal catalysts, which accounts for the differences in the graphitization degree of the obtained CNTs formed using different transition metal catalysts. Catalysts also assist in the decomposition of unsaturated hydrocarbon pyrolysis gases, such as dienes and alkynes in plastics, forming carbon atoms; hence, they assist in bond-breaking and chain-scission reactions during plastics degradation [55].

Acomb and co-workers [56] investigated the performance of Ni, Co, Fe, and Cu metal catalysts supported on alumina towards the growth of CNTs from LDPE feedstock using a two-stage pyrolytic-catalytic system at 600 °C and 800 °C pyrolysis and decomposition temperatures, respectively. They reported that Fe and Ni-based catalysts gave the highest CNTs yields of 179 mg CNTs/g plastic and 45.6 mg CNTs/g plastics, respectively. The high yield was attributed to the not-too-strong nor too-weak metal-support interaction and the high carbon solubility observed in the Fe/Al_2_O_3_ and Ni/Al_2_O_3_ catalysts. Cobalt-based catalysts resulted in low-yield CNTs of about 6 mg/g plastic due to the strong metal-support interaction. 10.13039/100014337Furthermore, Cu-based catalysts gave little or no CNTs as a result of the very weak interaction between the alumina support and Cu metal, which inhibited CNTs growth. Report by Nahil et al. [57] on the effect of incorporating various metals (Mg, Mn, Ca, Zn, or Ce) into nickel-based catalyst (Ni-Metal-Al), which were applied in the synthesis of CNTs from the pyrolysis-catalytic gasification of waste PP, showed that the highest CNTs yield was obtained with Ni–Mn–Al. This improved yield of filamentous carbon was attributed to the weak metal-support interaction between the Ni–Mn–Al catalyst. The order of catalysts showing the highest carbon deposition was Ni–Mn–Al > Ni–Ca–Al > Ni–Zn–Al > Ni– Ce–Al > Ni–Mg–Al. In a study by Guo et al. [58], the performance of Ni and Fe catalysts employed during the catalytic decomposition of phenolic formaldehyde resin into value-added CNTs was reported. They observed that CNTs deposited over Ni catalysts showed a less smooth structure with unreacted metal particles, while CNTs deposited over Fe catalysts were smooth with a distinct internal structure. Fe catalysts resulted in the formation of CNTs of higher yield and purity compared to Ni catalysts. The yield and purity of CNTs deposited over Fe catalysts were 34.39 % and 97.45 %, whereas Ni gave 24.07 % and 93.13 %, respectively.

Studies have also shown that bimetallic metal catalysts are more effective than monometallic catalysts in the growth of CNTs. Aboul-Enein and Awadallah [59] investigated the influence of adding Cu to Ni on the yield and nature of deposited carbon nanomaterials. CNMs were synthesized via a two-stage process at 500 °C and 700 °C pyrolysis and catalytic decomposition temperatures, respectively, over La_2_O_3_-supported Ni and Ni–Cu catalysts using waste PP plastic as a carbon precursor for 150 min. Their result showed that enhanced catalytic growth activity was observed for the bimetallic Ni–Cu/La_2_O_3_ catalyst, which displayed good metal dispersion, superior surface area, and a lower reduction temperature, unlike the monometallic 50%Ni/La_2_O_3_ catalyst, resulting in an improved CNM yield of 1458 % compared to the 944 % obtained over the Ni/La_2_O_3_ catalyst. A mixture of MWCNTs and large-diameter carbon nanofibers (CNFs) was deposited over a 40 % Ni–10%Cu/La_2_O_3_ catalyst, whereas only uniform-diameter MWCNTs were grown over 50%Ni/La_2_O_3_ catalyst. Their result invariably showed that the presence of La_2_O_3_ assisted in preventing Ni particle agglomeration while incorporating Cu into the Ni/La_2_O_3_ catalyst improved the catalytic decomposition activity. Recently, He and coworkers [60] demonstrated the influence of Mn addition as a promoter in enhancing the catalytic performance of Fe-based catalysts for CNTs production from the catalytic pyrolysis of PP. The effect of the addition of different Mn loading (0 wt%, 1 wt%, 5 wt%, and 10 wt%) to the Fe/Al_2_O_3_ catalyst was explored. They reported that increasing the Mn content in the Fe/Al_2_O_3_ catalyst enhanced the metal-support interaction due to the good active metal dispersion which hinders the sintering of Fe nanoparticles, resulting in better catalytic performance towards deposition of higher yield and more graphitized and quality CNTs. Overall, the catalyst with Mn loading of 5 wt% gave the best catalytic performance. However, increasing the Mn content up to 10 wt% resulted in the formation of the highest CNTs yield (about 41.53 %) but low-quality CNTs. Other cocatalysts, such as activated carbon (AC), have been utilized in the catalytic conversion of plastics into CNTs. Gong et al. [61] studied the production of CNTs using PP as carbon feedstock in the one-pot carbonization of waste PP using combined catalysts of AC/Ni_2_O_3_. They report that AC assisted in the dehydrogenation and aromatization of intermediates and light hydrocarbons to form CNTs. Short-length nanotubes with diameters in the range of 10–40 nm were obtained. The catalytic interaction between AC and Ni_2_O_3_ on the conversion of PP to CNTs was attributed to the presence of surface functional groups in AC.

### 2.2 Effect of catalyst composition on CNT synthesis

Yao et al. [62] studied the influence of Ni–Fe composition on the yield and quality of CNTs produced from the pyrolysis-catalysis of mixed plastics. Their study revealed that Ni and Fe composition influence the reactivity of the catalyst with Ni and Fe demonstrating different responsiveness for CNTs growth. According to their report, catalysts with the Ni:Fe ratio of 1:3 gave the highest CNT yield of 84.72 mg/g plastics. A higher Ni ratio in the Ni–Fe catalyst enhanced the graphitization degree and stability of synthesized nanotubes, while a higher Fe content in the Ni–Fe catalyst resulted in a higher carbon yield because of the weak interaction between the active metal catalyst and support. Aboul-Enein and Awadallah [63] studied several Fe–Mo/MgO catalysts with numerous Fe/Mo weight ratios (1.0, 0.9, 0.8, 0.6, 0.4, and 0.2), which were employed in the production of carbon nanomaterials from the catalytic-pyrolysis of waste LDPE. They reported that the morphological structure, yield, and the type of nanomaterial deposited on the surface of the FeMo/MgO catalyst could be manipulated by varying the Fe/Mo ratio. Their result revealed that higher Fe or Mo content resulted in the formation of CNTs and CNFs, while intermediate Fe/Mo ratios produced a mixture of CNFs, CNTs, and graphene nanosheets (GNSs). The highest CNMs yield of 976 % and 880 % were obtained over FeMo_(0.6)_/MgO and FeMo_(0.8)_/MgO catalysts, respectively. Less catalytic activity was obtained over Fe/Mo_(1.0)_/MgO compared to other Mo-containing catalysts due to the agglomeration of un-interacted Fe nanoparticles on the surfaces of MgO support, resulting in reduced metal dispersion and the number of active sites available for nucleation and growth of CNMs. The high catalytic activity of bimetallic Fe–Mo based catalysts of various Fe/Mo ratios were attributed to the improved metal particle dispersion and stabilization. Aboul-Enein and Awadallah [64] in their later study investigated the influence of adding various molar ratios of Co/Mo (14.5, 6.5, 2.5, 1, and 0.4) on the yield, morphology, stability, and quality (purity) of MWCNT produced using non-condensable gaseous feedstocks obtained from the catalytic degradation of LDPE using HZSM-5 in a two-stage pyrolysis-catalytic process over CoMo/MgO catalyst. They reported that the structure, purity, and quality of as-produced MWCNTs were strongly linked to the catalyst composition, where increasing the Co/Mo ratio in CoMo_(14.5)_/MgO resulted in the formation of high-purity MWCNTs. At a lower Co/Mo ratio, Co/Mo_(0.4)_/MgO resulted in a mixture of carbon nanofibers (CNFs), carbon nano-onions (CNOs) and MWCNTs. The highest CNT yield (1040 wt%) was obtained over the CoMo/MgO catalyst with Co/Mo molar ratio of 6.5. The authors also reported that decreasing Co/Mo ratio from 14.5 to 0.4 resulted in an increased CNT average diameter. Hence, a small quantity of Mo metal added to Co improved the catalytic activity of CoMo/MgO catalysts towards high-yield MWCNT grown from plastics. Bajad and co-workers [65] optimized molar ratios of Ni, Mo, and MgO contents in the NiMo/MgO catalyst during the combustion of waste PP for the synthesis of MWCNT. Other process parameters optimized in their study were combustion temperature, time, feedstock to catalyst weight. They reported that varying Ni/Mo ratios in NiMo/MgO catalysts impact nanotubes yield and diameter. According to them, increasing the Ni/Mo molar ratio from 0.5 to 20 resulted in the formation of small inner-diameter CNTs from 25 nm to 2 nm. Furthermore, lower Mo loading resulted in smaller diameter and high yield CNTs while higher Mo loading resulted in large diameter CNT. Overall, their study suggested an optimal yield of 514 % with 5 g waste PP combusted in 150 mg of Ni_4_Mo_0.2_MgO_1_ catalyst at 800 °C for 60 s.

### 2.3 Catalyst support

Several supporting materials (CaO, MgO, SiO_2_, Al_2_O_3_, TiO_2_, CaTiO_3_ (CaO–TiO_2_), CaO–SiO_2_, etc.) have been utilized in CNTs synthesis and added to active metal catalyst to precisely control and improve porosity, dispersion, inhibit subsurface diffusion and Ostwald ripening of active metal nanoparticles, etc. [52,66]. The size of catalysts’ active metals could be effectively controlled by the choice of suitable catalyst support, which invariably determines CNTs diameters [67]. The choice of appropriate support is vital for the catalytic performance, activity and stability of the catalysts in CNTs growth. Not too strong metal-support interaction results in better resistance to active metal catalyst sintering activity and also improves metal dispersion, considering its high tendency to coking and deactivation [50].

Yao et al. [68] studied the activities of four (4) support materials: MCM41, ZSM5, H-Beta, and NKF5 with different silica-alumina ratios on Ni–Fe catalysts during the decomposition of plastics pyrolysis volatiles obtained from mixed waste plastics to produce MWCNTs using two-stage pyrolysis-catalysis process. All synthesized catalysts showed different properties and different catalytic activity during the synthesis and growth of MWCNTs. The highest CNTs yield, and purity were obtained with Ni–Fe/MCM41, which displayed copious mesoporous structure and the highest surface area, resulting in good metal-support interaction and reducibility of the catalysts, while Ni–Fe/Beta gave the least graphitized CNTs. The catalytic activity for production of CNTs was in the following order: Ni–Fe/MCM41 > Ni–Fe/ZSM5 > Ni–Fe/Beta > Ni–Fe/NKF5. Their study suggested that the characteristics of the metal catalysts, and support material, specifically the metal dispersion, metal-support interaction, and the active metal composition had a great influence on the quantity and quality of the obtained CNMs. Jia and co-workers [69] demonstrated that variation in selectivity, yield, and structural properties of CNTs obtained from LDPE and PP is dependent on the degree of interaction between the supporting material and parent metallic catalyst. They employed Ni catalyst supported on different oxides of Sr, La, and Mg as a catalyst for the in-situ transformation of LDPE and PP into CNTs. According to their study, all catalysts (Ni/La, Ni/Sr, and Ni/Mg) exhibited different interactions between the Ni metal catalyst and support. Strong interaction in the Ni/Mg catalyst was not beneficial for CNTs growth. Moderate metal-support interaction in the Ni/La catalyst was favourable for the size and surface distribution of Ni nanoparticles, resulting in optimum catalytic activity with less defective CNT and improved yield of 114 % and 110 % from LDPE and PP feedstocks, respectively. The weak metal support interaction in Ni/Sr led to the combined growth of carbon nano-onions (CNOs) and few CNTs. The catalytic activity of the Ni catalyst increased in the following order: Ni/Mg < Ni/Sr < Ni/La. Shen and co-workers [70] suggested that the addition of Al to Ni catalyst resulted in a bimetallic catalyst with outstanding properties, increased surface area, improved stability, and decreased Ni particle sizes, which resulted in small-diameter CNTs. They reported that the highest CNTs yield of 85.5 % was obtained with the addition of 10 % Al, while the monometallic Ni catalyst gave a CNTs yield of 37.5 % from waste PP. According to their study, Ni/Al composition influenced the yield, morphology, and graphitization degree of CNTs, while higher compositions of Al resulted in reduced yield and quality of the obtained CNTs. Song et al. [71] also explored the effect of combining Ni_2_O_3_ with three different aromatization catalysts (H-beta, HZSM-5 zeolite, and OMMT) on the formation of CNTs from PP using the combustion technique. The highest CNTs yield was obtained with H-beta/Ni_2_O_3_ and OMMT catalysts compared to the HSM-5 catalyst. H-beta zeolite was demonstrated to be an effective catalyst in catalyzing the degradation of PP at high temperatures to produce CNTs. The highest CNTs yield and purity of 52 % and 90 %, respectively, were obtained from PP/H-beta/Ni_2_O_3_ composite when the content of H-beta was increased up to 5 %. Similarly, Veksha et al. [72] utilized nickel supported on different calcium supports (CaCO_3_, Ca(10.13039/100002264OH)_2_, CaO) and their mixtures for the catalytic degradation of non-condensable pyrolytic gases emitted from mixed plastics. The effect of different calcium supports and their mixtures on the performance (catalytic activity) of Ni-based catalysts during the hydrocarbon decomposition and HCl sorption from the non-condensable pyrolysis gas was demonstrated. They found that, to a large extent, the choice of support had a significant effect on the type, morphology, quality, and yield of deposited carbon on the surfaces of catalysts. Carbon deposited on the surfaces of Ni-supported CaO catalyst and mixtures were mostly of a non-filamentous structure while those Ni-supported Ca(10.13039/100002264OH)_2_ and CaCO_3_ catalyst were MWCNTs. Other supporting materials, such as anodic aluminium oxide (AAO) membrane [73] have been employed in growing CNTs from plastics.

### 2.4 Effect of catalyst preparation methods

Catalyst structure, composition and pore size can be tailored during its preparation. Synthesis of CNTs requires that carbon atoms diffuse into the catalyst's active site, resulting in supersaturation of the carbon in the metal and subsequent growth of CNTs [52]. The higher the number of active sites available, the higher the activity per site [52]. Control over the number and nature of active sites (active metals) is achieved during the catalyst preparation phases [74]. Numerous studies have been carried out on several catalyst preparation techniques viable for good activity and selectivity towards high-quality and high-yield CNTs. There are different catalyst preparation techniques employed in the dispersion of active species for CNTs growth, namely, chemical, physical vapour deposition (thermal evaporation), and sputtering methods [75,76]. This study focuses more on the preparation of catalysts via chemical techniques for CNTs growth from plastics. Procedures such as sol-gel, impregnation (wet and incipient wet), co-precipitation or precipitation, polyol, citrate methods, etc. have been extensively studied. Amongst the various methods, the dominant techniques mostly employed in catalysts' preparation during CNTs synthesis are sol-gel [77,78] and impregnation [79,80] techniques. Some of the experimental studies are provided towards the end of this section.

#### 2.4.1 Impregnation

In impregnation, the solution containing the catalyst precursor (active phase) is contacted with the support, and the obtained mixture is dried to remove the imbibed excess solvent and finally activated by calcination, and/or reduction treatment as may be required. The impregnation method is grouped into wet and incipient wet (dry or capillary) impregnation. In the wet impregnation method, an excess solution of the catalyst precursor is used. However, more control is achieved over incipient wet Impregnation (dry or capillary impregnation; here, the quantity of the solution bearing the catalyst precursor of appropriate concentration is contacted with support of an equivalent total known pore volume or, to some extent less [49,81].

#### 2.4.2 Precipitation or co-precipitation

This technique is employed in preparing bulk and supported catalysts. The choice of this procedure is preferred for active catalyst loading above 10–20 % [49]. Here, aqueous phase metal salts or active phase precursor is contacted with alkali solutions to form an insoluble metal hydroxide, and/or carbonates and subsequently filtered and dried to obtain solid mass which is usually ground to powder and further activated to convert the formed active hydroxides or carbonates, or nitrates into active oxides. Precipitation procedures are usually induced by variations in conditions such as pH, temperature, salt concentration, and rate of evaporation [49,82].

#### 2.4.3 Sol-gel

Sol-gel technique is also a chemical preparation method that involves creating solid materials from small molecular precursors. The solution called sol slowly results in the formation of a gel-like phase containing both liquid and solid phases. This procedure results in the formation of extremely monodispersed nanoparticles with a better degree of metal dispersion [83]. Furthermore, the sol-gel procedure offers improved control over the surface properties (surface area, pore size, and pore volume) of the catalytic material [75,[83], [84], [85], [86]]. Generally, the sol-gel technique is employed in CNTs synthesis due to the excellent ability to form oxides of the precursor metals and, at the same time, retain different structures inside the main structure even at high temperatures [75].

#### 2.4.4 Polyol method

This method involves the reduction of metal precursor salt in the presence of an appropriate structure-directing agent or capping agent such as polyvinylpyrrolidone (PVP) at high temperatures [87]. Typically, metal precursor salts are mixed with ethylene glycol (polyol). This method results in highly uniform and well-defined metal nanoparticles with different sizes, compositions, morphology, and crystallinity which are dependent on the polyol solvent, PVP, and metal precursor used.

Other techniques, such as the combustion synthesis method, have been utilized in metal nanoparticle preparation for CNTs synthesis [87].

In addition to these methods discussed above, catalyst preparation by the citrate method is a type of sol-gel method where citric acid is used as a complexing agent [75,84]. Apart from citric acid, other organic additives (dispersants) such as poly-(ethylene glycol) 10.13039/501100010368PEG [88], ethoxylated sorbitan ester [89], etc. are employed during catalyst preparation as complexing- and/or structure-directing agents in order to obtain an improved specific surface area of the support, uniform, and well distributed active metal nanoparticle phases. Hence, enhances the interaction between the catalyst and support (metal-support interaction) which also influences the yield of CNTs during synthesis [88].

The preferred catalyst preparation technique depends on the required physical and chemical features of the catalyst nanoparticles. Several comparison investigations have been carried out on the influence of different catalyst preparation techniques on the deposition and growth of CNTs from plastics. Modekwe et al. [90] investigated the catalytic performance of NiMo/MgO catalyst prepared using two different catalyst preparation techniques: incipient wet impregnation and sol-gel methods, during the catalytic growth of CNTs from waste PP plastics. They reported that catalyst porosity, degree of active metal (Ni) dispersion, crystallite size, and performance in terms of the yield of carbon deposited on the surface of the catalyst are linked to the catalyst preparation technique. Their result showed that optimal dispersion and homogeneity were obtained in catalyst materials prepared using the sol-gel method; however, incipient wet impregnated catalyst grew the highest CNTs yield. Similarly, Yang et al. [91] shed more light on the effect of two catalyst preparation techniques, impregnation and polyol procedures on the performance of the Ni/Al-SBA-15 catalyst employed in the growth of CNTs during the gasification of waste PP and PE plastics. They reported that the catalytic performance of the catalyst loaded with 10 wt% Ni on Al-SBA-15 with a Si/Al molar ratio of 10 (i.e., 10Ni/Al-SBA-15) prepared using polyol procedure produced additional strong acidic sites and mesoporous hexagonal structure, which stimulated the dehydrogenation and aromatization of waste PP and PE plastics resulting in higher yield and quality CNTs with uniform diameter. Yao and Wang [92] also utilized impregnation and sol-gel techniques in the preparation of several Fe/Ni catalysts, which were utilized in the pyrolysis and catalytic decomposition of PP to produce CNMs and hydrogen. Their studies showed that sol-gel prepared catalysts were more effective than impregnated catalysts during the catalytic decomposition of PP, resulting in the formation of highly graphitized and small-diameter CNTs. This result, according to them, was attributed to the formation of a uniform mesoporous structure with a high specific surface area of 212.30 m^2^/g, high active metal dispersion, and enhanced reducibility obtained from sol-gel prepared Fe/Ni catalysts was attributed to the good synergism between Ni and Fe. Also, in another study by Yao and co-workers [93], the authors reported higher catalytic activity of sol-gel prepared FeNi catalyst towards the growth of high quality, uniform diameter, and few defect-density nanotubes. FeNi catalyst prepared via the sol-gel method showed higher and better performance than similar FeNi catalyst prepared by conventional impregnation techniques when utilized in the catalytic decomposition of pyrolytic hydrocarbon gases obtained from waste PP for CNTs synthesis.

### 2.5 Catalyst pre-treatment conditions

Other conditions such as gas environment, calcination, and reduction temperatures have been studied to influence the structure, surface properties, and activities of catalysts utilized during CNTs nucleation and growth. Thermal treatments of the catalyst, such as catalyst calcination and reduction have a distinct impact on the activity of catalysts [49]. Calcination is very crucial in regulating the size of nanoparticles and the interaction between metal nanoparticles and support, which influence the overall catalyst activity and stability [56,94]. High calcination temperature could collapse the wall porous structure of catalyst material, lowering their total surface area and consequently resulting in porosity breakdown, which could lead to their agglomerations and larger particle size [94]. Reduction of the catalyst in hydrogen or other reducing agents removes the oxygen lattice in the catalyst which resulted in the emergence of active metal sites, at which the nanotubes could grow. Likewise, excessive reduction exposes metallic catalysts as they get agglomerated even before CNTs nucleation and growth, resulting in poor quality CNTs with larger diameter distribution [95]. Modekwe et al. [96] reported the effect of different calcination temperatures (600, 700, and 800 °C) on the NiMo/CaTiO_3_ catalyst on the yield, morphology, and quality of CNTs produced from the catalytic decomposition of waste PP. Their study revealed that as catalysts’ calcination temperatures increased from 600 to 800 °C, the porous wall structure of catalyst material gradually collapsed, resulting in a continuous decrease in total surface area and pore volume of catalysts. Also, an increase in calcination temperature resulted in the emergence of more metastable phases and improved metal-support interaction. According to their study, an increase in thermal treatment from 600 to 800 °C had negligible influence on CNTs yield. Thermal treatment of the catalyst calcined at 700 °C produced the best quality, structurally ordered, long- and small-diameter CNTs. Liu et al. [97] reported the formation of Ni/SiO_2_ and Fe/SiO_2_ catalysts prepared by sol-gel technique with different metal particle sizes were when activated (calcined) under different gas environments. Calcination in N_2_ and air environments resulted in formation of small and large metal particles sizes, respectively. Their study revealed that metal particle sizes of Fe–SiO_2_ and Ni–SiO_2_ influenced the catalyst performance towards CNTs formation by catalytic gasification of waste PP using a two-stage fixed-bed reactor process. Their result showed that both Fe- and Ni-based catalysts with large particle sizes (Fe/SiO_2_-L and Ni/SiO_2_-L) gave highest quality and yield CNTs (29 wt% and 16 wt%, respectively) whereas Ni-based catalyst with small metal particle size about 8 nm (Ni/SiO_2_–S) resulted in the formation of huge quantities of amorphous carbon. Hence, not suitable for CNTs nucleation and growth. Therefore, activation under different carrier gases has different effects on catalyst properties and CNTs structure.

## 3 Effect of temperature

### 3.1 Pyrolysis temperature

Thermal degradation of polymers (plastics) is a complex process requiring heat and mass transfer. The decomposition behaviour of plastic materials is controlled by temperature since different materials have different optimal decomposition temperatures. During plastics decomposition, the van der Waals forces between the walls of the molecules collapse because of the increased collision frequency of the hydrocarbon molecules, leading to the breaking of individual carbon chains; hence, particles at the surface vaporize while bonds are being broken [12]. According to studies in the open literature, higher pyrolysis temperatures increase the gaseous product concentrations [[98], [99], [100]]. Aboul-Enein et al. [79] demonstrated the effect of degrading waste LDPE at different pyrolysis temperatures (500, 600, 700, and 800 °C) on the yield and morphology of CNTs synthesized at 700 °C over a 10%Ni–Mo/Al_2_O_3_ catalyst. According to their findings, improved CNTs yields from 14.7 % to 27.8 % were observed as the degradation temperatures were increased from 500 °C to 700 °C, respectively. Further increase in temperature up to 800 °C resulted in a marginal difference in CNTs yield (28.1 %). Such an increase in CNTs yield was claimed to be due to a corresponding increase in the volume of obtained gaseous fractions as the temperature was increased from 500 to 700 °C. Their study also noted that higher pyrolysis temperature resulted in the formation of uniform diameter CNTs. Also, Bajad and co-workers [101] studied the influence of pyrolysis temperature of HDPE at 450–700 °C on the yield and morphology of CNTs produced over Ni/Mo/MgO catalyst using a multi-core batch reactor. They found that the optimal CNTs yield of 6.033 g/30 g HDPE was obtained at 700 °C pyrolysis temperature, with the lowest yield obtained at 450 °C pyrolysis temperature and 800 °C synthesis temperature. Higher pyrolysis temperatures induced an increase in gas yield, promoting the formation of a wide range of smaller organic molecules, which are a better feedstock for CNTs growth. Liu et al. [78] demonstrated the influence of pyrolysis temperature (550–750 °C) and decomposition temperature (500–800 °C) on the yield of CNTs obtained from the catalytic degradation of PP in HZSM-5 zeolite catalyst and subsequent decomposition of obtained pyrolytic gases over NiO catalyst in a moving-bed reactor. From their result, increasing the pyrolysis temperature from 550 to 750 °C enhanced the volume of obtained gaseous products from 74.9 to 119.1 L/100 g PP. According to their report, a high synthesis temperature resulted in the growth of well-graphitized and thermally stable CNTs. In terms of CNTs yield, they found that decomposition temperatures from 500 to 700 °C resulted in an improved yield of 23.7–34.1 g CNTs/100 g PP, while further increase up to 800 °C reduced the yield to 32.2 g CNTs/100 g PP. Yang et al. [102] reported that increasing the fluidized bed temperature from 500 to 700 °C favoured plastic decomposition and degradation of larger molecules into smaller hydrocarbon molecules, which resulted in an increased concentration of CO_2_, H_2_ and CH_4_ during the gasification of waste plastics (PP and PE) in a fluidized bed reactor. The dominance of light hydrocarbon products favoured CNTs yield. Subsequent catalytic reforming and cracking of obtained light hydrocarbon products over Ni/Al_2_O_3_ in a fixed bed reactor at 680 °C resulted in improved CNTs yield and quality.

### 3.2 Synthesis/decomposition temperature

The growth of CNTs follows that carbon atoms from the dissociated hydrocarbons must first dissolve into the catalytic metal sites, and then the diffused carbon atoms precipitate and form graphitic sheets on the surface of the metal particles [93,94]. Hence, the rate of diffusion of carbon atoms is a key determining factor in the nucleation and growth of CNTs. Therefore, increasing temperature can stimulate the diffusion rate of carbon atoms in the metal catalysts, which could result in improved yield with fewer defective CNTs.

Nevertheless, at an extremely high synthesis temperature, sintering and agglomeration of catalytic sites may occur due to the formation of larger catalyst particles, since an increase in temperature intensifies collisions in the gas phase, resulting in the deactivation of catalysts. In addition, excessive accumulation of carbon atoms on the catalyst surface could encapsulate catalytic sites, triggering catalyst deactivation [52]. Liu et al. [103] reported the influence of temperature, Ni content, and water injection on the growth of CNTs from HDPE deposited over Ni/anodic aluminium oxide in a two-stage fixed-bed reactor. According to their study, 700 °C was the ideal temperature for the formation of high-yield CNTs, while 600 and 800 °C resulted in the formation of low-yield CNTs. Steam injection impacted the yield and quality of grown CNTs over Ni/AAO. In another study by Liu et al. [104], the authors suggested that the effect of reaction temperature on CNTs synthesis by CVD was mainly related to carbon source and catalytic sites. In their study, they investigated the effect of reaction temperatures (600–800 °C) and Ni loading on CNTs grown from HDPE as feedstock in the presence of Ni-based ceramic membrane catalyst (Ni/Al_2_O_3_) using a two-stage reactor. An increase in Ni content on ceramic membranes resulted in increased diameters of metal particle sizes, which affected the activity of catalysts and CNTs formation. 1.0/ceramic was the optimum loading and at 700 °C, the optimal amount of filamentous carbon was obtained. At 800 °C, only a few filamentous carbon and excessive amorphous carbon were observed. Zhang et al. [105] also reported the influence of different catalyst temperatures (700, 800, and 900 °C) on the growth of CNTs from pyrolysis-catalysis of HDPE over Ni-stainless-steel mesh catalyst. Their result showed that the highest yield of carbon deposit was obtained at 900 °C while the most graphitic carbon was obtained at 800 °C catalysis temperature. Again, the diameter of filamentous carbon (CNTs) obtained at 700 °C was smaller compared to those produced at higher catalyst temperatures. Zheng et al. [106] reported the effect of catalysis-decomposition temperatures; 500, 600, 700, and 800 °C, on CNTs yield obtained from the NiCl_3_ and waste PE composite. Their study revealed that the highest carbon yield of 17 wt% was obtained at a catalysis-decomposition temperature of 700 °C with a catalyst-feedstock ratio of 0.75 wt%, while a further increase to 800 °C resulted in a reduced yield, signifying that a higher temperature was not suitable for CNTs growth. However, increased temperatures up to 800 °C favoured the graphitization degree of produced CNTs. Liu et al. [107] studied the effect of reaction temperatures of 600, 700, and 800 °C on the formation of CNTs deposited on the surfaces of Ni/spherical alumina support catalyst during the catalytic thermo-chemical conversion of HDPE. Their result showed that at 600 °C, only amorphous carbon was observed with no CNTs; increasing the temperature up to 700 °C, a few CNTs and amorphous carbon were seen. At 800 °C growth temperature, numerous uniform diameters and well-aligned CNTs were observed. As the synthesis temperature increased from 700 to 800 °C, the yield of CNTs increased from 1.0 wt% to 7.5 wt% and the quality of the as-obtained CNTs was improved. In a study by Tripathi et al. [108] temperature parameter was optimized (600, 700, 800, 900, 1000, and 1100 °C), and its effect on the quantity and quality of grown MWCNTs from waste PP using a two-stage stainless-steel (SS 316) tube CVD reactor was reported. Waste PP was pyrolyzed at 10 °C/min in the first reactor at 500 °C under a 100 cm^3^/min Ar gas atmosphere, while the second (stainless-steel tube, SS 316) reactor functioned as both a catalyst and a reactor unit for the catalytic decomposition of produced hydrocarbon gases and subsequent synthesis of nanotubes. They found that under oxidized SS 316 reactor condition, the highest yield (42 %), well graphitized with less defective (I_D_/I_G_ = 0.48) MWCNTs was obtained at 900 °C. Their study showed that under different reaction temperatures, different carbon structures (amorphous carbon, carbon nanofibers, MWCNTs, soot, spheres, etc.), morphology, quality and yield were formed.

## 4 Effect of different plastics feedstock

CNTs production from plastics has been shown to depend on its composition and the concentration of carbonaceous gases released during degradation. Different types of feed result in variations in yield, structure, and even quality of CNTs [109]. Several studies in the open literature have extensively investigated the activities of different plastic materials as a factor in the production of better quality and high-yield CNTs. Different plastic materials such as PE, PVC, PS, PP, and PET have been adopted for CNTs synthesis and have demonstrated certain structural variations from various plastics due to variations in the composition and the ease of release of their pyrolysis products (gases, liquid, and wax). Pyrolysis products of polyolefins are mainly methane, ethylene, ethane, propane, i-butylene, i- & n-butene, n-paraffine, vinyl olefin, etc., while PVC is mostly composed of more complex products with HCl, benzene, alkyl aromatics, alkanes, wax, etc [110]. Chloride element in PVC [109] could bond with carbon or hydrogen atoms, resulting in the poisoning of metal catalyst particles during CNTs synthesis. Hence, is unfavourable for the formation of high-yield and low-defect-density CNTs. Waste plastics contain impurities such as dirt, additives, colourants, fillers, etc. The presence of these materials in the feedstock used in CNTs synthesis may significantly affect the quality as well as quantity of the CNTs produced [22,111]. Plastic waste materials are preliminarily pretreated before their usage in CNTs synthesis to remove impurities such as dirt. This is achieved by simply washing and rinsing using different chemicals such as soap solution and deionized water [41], anhydrous alcohol (ethanol), acetone [112], etc.

Aboul-Enein and co-workers [113] were able to link the amount of hydrocarbon gaseous products produced from the thermal decomposition of different types of plastic wastes to the yield of CNTs. In their study, polyolefins (such as PP, LDPE, and HDPE), PS, and PET were utilized as feedstocks for producing MWCNTs over NiMo/Al_2_O_3_ catalyst using a two-stage CVD method. They reported that the highest gaseous products were obtained from polyolefins; LDPE and PP with 72.5 wt% and 70.7 wt%, respectively. PP gave the maximum CNTs yield of up to 5.8 g/g catalyst while LDPE produced 5.0 g/g catalyst, whereas utilizing PS and PET resulted in the formation of poor yield and very low-quality CNTs, making them an inappropriate feedstock for CNTs synthesis. In a study by Panahi et al. [114], the yield and purity of CNTs obtained from polymer feedstocks were found to strongly depend on the type of plastic feedstock. According to their study, the order of highest CNTs yield according to polymer feedstock under similar conditions and catalysts was PP > PE > PS > PET. Similarly, CNTs’ length, wall thickness, and diameter were also reported to strongly depend on the type of plastic feedstock used, type of catalyst substrate, and catalyst pre-treatment method employed during catalyst preparation. Veksha, and co-workers [80] also reported the influence of different plastics feedstocks (PP, LDPE, MP, and PET) on the yield, graphitization degree, and type of carbon nanomaterials produced over Ni catalyst at two different synthesis temperatures of 500 and 800 °C. According to their study, at 500 °C, MP produced a mixture of carbon nanocage and MWCNTs with a very low yield (3 %) while LDPE and PP resulted in mainly MWCNTs with a yield of 21 % and 32 %, respectively. Carbon materials produced at 800 °C were all MWCNTs with similar yield, quality, and structure for all plastic feedstocks used. According to the authors, these results could be due to the differences in the concentration of unsaturated hydrocarbons present in pyrolysis gases released from different plastics at different temperatures. Cai et al. [115] demonstrated the connection between the different pyrolysis products (gaseous, liquid, and carbon deposition) released from the different plastic types to the yield and properties of deposited filamentous carbon (CNTs) over Fe/Al_2_O_3_ catalyst. Different polymers such as PP, PE (LDPE and HDPE) and PS (high impact polystyrene, HIPS and general-purpose polystyrene, GPPS) were investigated. Polyolefin wastes showed highest yield of gaseous products; a large yield of carbon deposits was obtained with GPPS and HIPS about 48.7 and 49.4 wt%, respectively. Despite the considerable amount of carbon deposited, PP, LDPE and HDPE produced a higher percentage (over 75 %) of graphitic carbon deposit compared to GPPS and HIPS, which had a 10 % higher proportion of amorphous carbon. They found that CNTs produced from the pyrolysis-catalysis of PP plastics gave the greatest degree of graphitization. Overall, CNTs obtained from PP and PE showed a higher degree of graphitization and improved yield compared to PS.

Table 1 summarizes results from several studies that employed different types of plastic materials as feedstock in the synthesis of CNTs under different synthesis conditions and the corresponding characteristics (quality, yield, and diameter) of produced plastic-derived nanomaterials.

**Table 1:** Summary of studies using different types of plastics as feedstock for synthesis of CNTs under different conditions.

Feedstock	Catalyst	Catalyst support	Catalyst composition/ratio	Catalyst preparation Method	Catalyst treatment condition	Reactor type	Synthesis Temperature	CNTs diameter	CNTs yield	CNTs quality	Ref.
PS	Ni–Mo Ni Ni–Mo Ni–Mg Ni–Mo–Mg	Mg	5:0:0 5:0.1:0 5:1:0 5:0.1:1	Combustion method	650 °C for 1 h	One-step carbonization in a quartz tube (fixed bed reactor)	1000 °C for 10 min	500–1000 nm 300–500 nm 40–50 nm 12–20 nm	21 wt% 19 wt% 35 wt% 42 wt%	– – – I_G_/I_D_ = 1.3	[116]
PP	Ni/Mo	MgO	5/0/0	Combustion method	650 °C for 1 h	Combusted using a crucible in a muffler furnace	850 °C for 10 min	–	5 %	–	[117]
			5/0.1/0					–	34 %	–	
			5/0.1/0.25					–	41 %	–	
			5/0.1/0.5					–	49 %	–	
			5/0.1/1.0					–	58 %	–	
			5/0.1/1.25					–	51 %	–	
			5/0.1/1.5					–	48 %	–	
PP + PE	Ni	Al_2_O_3_	10 wt% Ni at 0.1 Equivalent Ratio	Impregnation method	500 °C for 3 h under Air, N_2_ and 5%H_2_/He	fluidized bed reactor	600 °C for 10 min	– – 12–20 nm	8.4 % 16.6 % 22.0 %	I_G_/I_D_ = 0.66 I_G_/I_D_ = 0.43 I_G_/I_D_ = 0.71	[118]
PP + PE	Ni	Al_2_O_3_	–	Impregnation	500 °C for 3 h under 5 % H_2_/He	Fluidized fixed bed reactor	At different fluidized bed temperatures: 500 °C 600 °C 700 °C	10–12 nm – 33 nm	– 22.0 % 25.8 %	I_G_/I_D_ = 0.65 – I_G_/I_D_ = 0.86	[102]
PE	Fe_2_O_3_	MgO	Fe:Mo weight ratio = 8.4 %	Impregnation	–	Chemical vapour deposition- fluidized bed reactor (CVD-FBR)	Upper/lower temperatures 750/850 °C Under: Ar 10%H_2_/Ar 20%H_2_/Ar	25–50 nm 25–50 nm 25–50 nm	– – –	I_D_/I_G_ = 0.79 I_D_/I_G_ = 0.67 I_D_/I_G_ = 0.60	[119]
PS	Ni–Mo Ni–Mo	MgO Mg/5 wt% CB	7 wt% Ni 7 wt% Ni & 5 wt% CB	Combustion	650 °C for 1 h	One-stage pyrolyzation in a furnace	850 °C for 15 min under N_2_	5–25 nm	18.39 %	I_G_/I_D_ = 0.99	[120]
PP								5–20 nm	26.24 %	I_G_/I_D_ = 0.79	
PE								5–25 nm	28.36 %	I_G_/I_D_ = 0.90	
PP + PE + PS								5–25 nm	31.66 %	–	
PS								5–25 nm	41.85 %	I_G_/I_D_ = 1.1	
PP								5–25 nm	53.56 %	I_G_/I_D_ = 1.07	
PE								5–25 nm	65.95 %	I_G_/I_D_ = 0.96	
PS + PP + PE								5–25 nm	59.83 %	–	
PE + MA-PP	Ferrocene					Stainless steel autoclave	700 °C for 1 h	20–60 nm	80 % with 5 % helical CNTs	I_D_/I_G_ ≥ 1	[121]
HDPE	Ni	AAO	10 wt%	Wet impregnation	700 °C for 3 h	Two-stage catalytic CVD	500 and 700 °C under N_2_	50–60 nm	–	–	[73]
PP	Ni	–	–	Urea decomposition method	–	Fixed bed reactor (Single-stage CVD chamber)	Under 10%H_2_/Ar 600 °C 700 °C 800 °C	– – 10–25 nm	– – 19 %	– I_G_/I_D_ = 0.63 I_G_/I_D_ = 1.20 I_G_/I_D_ = 1.74	[122]
Waste LDPE	Co	MgO	–	Impregnation	–	Fixed bed quartz reactor	550 °C for 1 h under H_2_/Ar	–	–	I_D_/I_G_ = 1.1	[123]
PP + LDPE	Ni Ni–Cu	Al-SBA-15-PL CaO–SiO_2_- PL (PL-powder)	10 wt%Ni	Polyol method	500 °C for 3 h under 5%H_2_/He	Two-stage fluidized-catalytic bed reactor	600 °C at 0.1 ER and 800 °C (2nd stage)	10–16 nm –	10–17 % 48 %	– –	[124]
HDPE + LDPE	Cobalt acetate	–	20 wt%	–	–	Autoclave batch reactor	700 °C	80 nm			[125]
LDPE HDPE	Ni_x_Fe_1-x_O_3-⸹_ H-LF H-LN_0.15_F_0.85_ H-LN_0.5_F_0.5_ H-LN_0.85_F_0.15_ H-LF H-LN_0.15_F_0.85_ H-LN_0.5_F_0.5_ H-LN_0.85_F_0.15_	La_0.8_	(x = 0, 0.15, 0.5, 0.85)	Sol-gel combustion method	300 °C for 1 h and 800 °C for 5 h under air	Two-stage thermo-catalytic process	1st = 700 °C 2nd = 800 °C	45 nm 44 nm	0.38 g/g 1.44 g/g 1.16 g/g 1.37 g/g 0.68 g/g 1.40 g/g 1.32 g/g 1.34 g/g	I_D_/I_G_ = 0.61 I_D_/I_G_ = 0.61 I_D_/I_G_ = 0.58 I_D_/I_G_ = 0.55 I_D_/I_G_ = 0.61 I_D_/I_G_ = 0.63 I_D_/I_G_ = 0.58 I_D_/I_G_ = 0.51	[126]
Mixed plastics (HDPE + PP + PS + PVC + PET)	Ni/Mo	MgO	4:0.1:1 (Ni:Mo:MgO)	Sol-gel method	700 °C for 2h	Multi-core reactor system	500–700 °C (pyrolysis)/700–900 °C (synth.) Temperatures 600/800 700/800 600/800 600/700 700/900	40–45 nm 20–25 nm 40–45 nm 40–45 nm 20–25 nm	1.66 wt% 6.63 wt% 1.47 wt% 3.13 wt% 5.87 wt%	I_G_/I_D_ = 1.04 I_G_/I_D_ = 1.13 I_G_/I_D_ = 1.03 I_G_/I_D_ = 0.88 I_G_/I_D_ = 1.40	[127]
PP	NiMo	CaTliO_3_	Molar ratio (Ni:Mo:CaTiO_3_) 4:1:2 4:1:4	Sol-gel method	700 °C for 3 h under air	Single-stage CVD (Fixed bed) reactor	700 °C	5–35 nm 12–36 nm	44 % 35 %	I_G_/I_D_ = 1.15 I_G_/I_D_ = 0.96	[96]
PP	Ni/Ca Ni/Zn	Al Al	20 wt% Ni	–	–	Two-step gasification reactor	800 °C	50 nm	0.053 g/g Catalyst 0.012 g/g catalyst	I_D_/I_G_ = 0.58 I_G'_/I_G_ 0.94	[128]
Real-world mixed plastics (HDPE + PP + LDPE + PS)	Ni–Fe Ni/α-Al_2_O_3_ Ni/γ-Al_2_O_3_ Fe/α-Al_2_O_3_ Fe/γ-Al_2_O_3_ Ni–Fe/γ-Al_2_O_3_	α-Al_2_O_3_ γ-Al_2_O_3_ – – – – –	1:3 (Ni to Fe molar ratio)	Impregnation technique	800 °C for 3 h under air	Two-stage fixed bed reactor	500 & 800 °C	40–50 nm 20 nm – – 20–40 nm	26.1 % 21.1 % 35.2 % 32.6 % 40.7 %	I_G'_/I_G_ = 0.21 I_G'_/I_G_ = 0.40 I_G'_/I_G_ = 0.58 I_G'_/I_G_ = 0.59 I_G'_/I_G_ = 0.65	[129]

## 5 Other conditions

### 5.1 Effect of feedstock to catalyst ratio

It is expected that the more hydrocarbon feedstock used, the larger the source of carbon, which would give rise to a larger amount of CNTs per gram of catalyst or plastics used. Generally, catalyst activity reduces as it becomes overloaded, which may trigger early catalyst deactivation, leading to a larger portion of the hydrocarbon gases left unreacted. Therefore, in terms of the percentage of feedstock converted into CNTs, a reduction could be observed. A great deal of studies have been done in this area to ascertain the optimal balance between catalyst activity and feedstock loading. Acomb et al. [130] studied the effect of feedstock-to-catalyst ratio on the growth of CNTs from LDPE over Fe/Al_2_O_4_ catalyst. They found that by increasing the amount of LDPE feedstock relative to the catalysts, the percentage conversion of plastics to CNTs was reduced. When 0.5 g of LDPE was used, 29.1 wt% of plastics was converted to CNTs. While increasing the loading of LDPE feedstock up to 1.25 g resulted in 13.1 wt% conversions with predominant growth of amorphous (non-crystalline) carbon. They reported that the reduction in the catalytic activity was due to the overloading of the catalyst surface, resulting in many unreacted hydrocarbon gases. An earlier study by Yang and co-workers [131] demonstrated that the diameter of CNTs could be precisely tailored by controlling the growth temperature and feeding rate of the catalyst precursor. During the synthesis of vertically aligned CNTs arrays from PP using ferrocene as catalyst in the floating catalyst CVD technique, their study showed that the length of CNT arrays increases linearly with growth time and feeding rate of catalyst precursor (0.1, 0.4, and 0.8 g/min) at a fixed feeding rate of 40 g/h of the carbon feedstock. For example, within 40 min, the average growth rate was about 12 μm/min, and the length of CNTs arrays reached 500 μm. The diameter distribution of all obtained well-ordered CNTs structures decreases with increasing feeding rates of catalyst precursors, from 36 nm to 22.6 nm. The purity of the CNTs arrays obtained from PP was 97.4 % with a low defect density. Zhang et al. [105] also demonstrated that increasing the HDPE plastic-to-catalyst (Ni-stainless-steel mesh) ratio impacted the yield of the obtained filamentous carbon. According to their study, increasing the plastic-to-catalyst ratio from 2:1 to 4:1 resulted in a reduction in the yield of filamentous carbon (MWCNT) from 38.0 to 25.7 wt%, equivalent to a reduction from 374.06 mg CNTs/g plastic to 247.03 mg CNTs/g plastic.

### 5.2 Effect of steam addition

Acomb et al. [132] reported the influence of various steam injection rates (0, 0.25, 1.90, and 4.74 g/h) on the quality (purity) of CNTs produced from a two-stage pyrolysis-gasification process of three different types of waste plastics, namely PS, PP and LDPE in the presence of Ni/Al_2_O_3_ catalysts at 600 and 800 °C pyrolysis and reformation temperatures, respectively. Their investigation revealed that by regulating the steam injection rate, the highest quality CNTs were obtained with different waste plastics at a low steam injection rate of 0–0.25 g/h. At a 0 g/h steam injection rate, high CNTs yield, and purity were obtained with LDPE. However, with PS and PP, the highest CNT yield was obtained at 0.25 g/h steam injection rate. Increasing the steam content in the gasification unit from 1.9 to 4.74 g/h is detrimental to the purity and yield of filamentous carbon. Wu et al. [133] also reported the influence of steam addition in the production of CNTs and H_2_ from waste HDPE and mixtures of HDPE and PVC feedstocks in a two-stage pyrolysis-reforming system over Ni–Mn–Al catalyst. Their study revealed that steam addition resulted in the formation of more defective CNTs with reduced total carbon yield. Also, the injection of steam resulted in less ordered and quality CNTs. Yao and co-workers' [129] findings also concluded that optimum CNTs yield was obtained in the absence of steam. Another study by Gogotsi and team [134] demonstrated that adding water during the hydrothermal growth of CNTs from PE resulted in the formation of hollow tubes, while in the absence of water, thick-walled tubes with several internal closures were formed.

### 5.3 Influence of deposition time and heating rate

Residence time is the average amount of time that material spends in the reactor, and it may affect product distribution and formation [17,110]. Longer residence time increases the conversion of primary product in the case of pyrolysis time in pyrolysis reactors, although there are instances where temperature limitations in the process may influence the overall product distribution, and the residence time may have less effect on the product distribution [17,135].

Little is reported in the open literature on how average durations for pyrolytic gases during catalytic deposition affect the overall CNTs growth process during synthesis from plastic.

In CNTs synthesis by CVD process, studies are carried out at atmospheric pressure and constant temperature. Based on the CVD growth mechanism, longer CNTs catalytic deposition times result in a longer constant diffusion reaction duration with increasing mass deposition over the catalyst surface [136]. Therefore, as deposition time increases, carbon atom diffusion and precipitation on the catalyst surfaces decrease due to increased wall thickness and reduced pore channels hindering carbon atoms passage and diffusion [136]. Hence, less deposition and growth rate, and consequently, low CNTs yield.

The heating rate is also another parameter of interest whose influence on the yield and quality of CNTs synthesized from plastics is rarely investigated. The rate at which plastic materials and pyrolytic gases that form the carbon atoms are heated during catalytic decomposition reaction may influence the overall yield and quality of produced CNTs [137]. A recent study by Eldahshory et al. [138] investigated the influence of various heating rates (5, 10, and 20 °C/min) on the yield and quality of CNTs synthesized from waste PP over nickel foam catalyst. Their study reported that increasing the heating rate from 5 to 20 °C/min resulted in the production of CNTs of reduced quality with a large wall thickness and diameter and a low yield. The authors further reported that the optimum heating rate and CVD temperature to achieve maximum CNTs quality and yield of 39.34 % were at 5 °C/min and 700 °C, respectively.

## 6 Reactor configuration, design, and synthesis techniques for CNTs growth from plastics

**Fig. 2:**
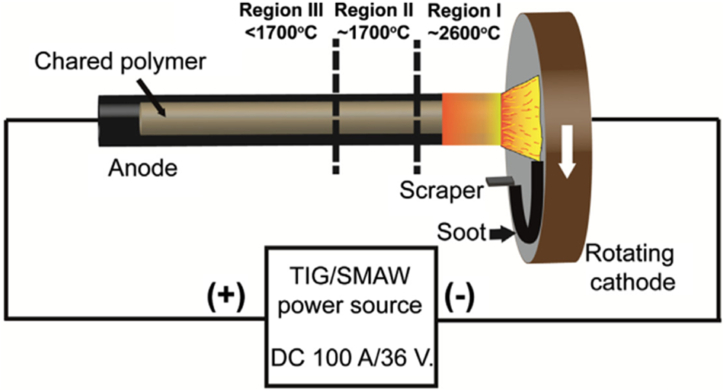
Schematic of rotating cathode arc discharge for nanomaterials synthesis from PET char. Adapted from Ref. [141] with permission from Elsevier.

The common methods employed in CNTs synthesis are arc discharge, chemical vapour deposition, laser ablation, flame synthesis, spray pyrolysis, etc. [35,39]. Electric-arc discharge is one of the earlier methods of producing CNTs. Here, a mixture of graphite powder and transition metals, which could be Ni, Co, or Fe, were loaded into a hollow graphite anode and vapourised by an electric arc at about 2000–3000 °C under an inert atmosphere maintained at 50–600 Torr [35,139]. The yield of arc-produced CNTs was usually around 30 % in short-structured tubes with an outer diameter of SWCNTs around 0.4–3 nm and MWCNTs of about 4–30 nm [139,140]. The impurity content obtained using this method is larger compared to other methods. Berkmans et al. [141] utilized the solvent- and catalyst-free rotating cathode arc discharge method to synthesize MWCNTs and nano-channeled ultrafine carbon tubes (NCUFCTs) from waste PET. According to their report, PET waste is first pyrolyzed in a stream of N_2_ at 815 °C for 20 min. The obtained char was ground and tightly packed into the hollow graphite anode (see Fig. 2), where it was vapourised by the cathode arc in nitrogen (N_2_) atmosphere at a pressure of 500 Torr for continuous synthesis for I min at 1700–3000 °C (depending on the region). Soot was collected at both the cathode disc and the tip of the anode. Soot obtained at the cathode is found to be more crystalline than that obtained at the anode. Anode soot contain ultrafine and nanosized solid carbon spheres of diameter 221 nm and 100 nm, respectively, and also tubular structures (MWCNTs) of diameter 95 nm. Soot deposited at the cathode disc is composed of mixtures of MWCNTs with a mean diameter of 20 nm and other nanoparticle impurities such as fullerenes, fused graphites, and other polyhedral nanoparticles.

In the CVD synthesis of CNTs, hydrocarbon molecules are decomposed into carbon species in their gaseous phase using an electrically heated furnace and deposited on top of heated catalyst material [36,48]. The high temperature decomposition reaction causes carbon atoms to reach the surface of the metal catalyst, diffuse into the catalyst, dissolve, and form a metal-carbon solution, which becomes supersaturated and carbon precipitates in the form of a nanotube [35,142]. During CVD synthesis, controlling any CVD process parameters (reaction temperature, gas flow rates, catalyst or substrate type or quantity, etc.) can lead to the formation of CNTs of different or desired characteristics in morphology, diameter, yield, and quality [143].

Amongst these methods, chemical vapour deposition (CVD) is the most promising and preferred technique for possible scale-up and large-scale production of CNTs at low cost due to its energy efficient, easy workability with any raw material (any hydrocarbon containing liquid, solid, or gas), ease of control over process parameters, high purity and yield, and ease of operation [38]. Forms of CVD techniques that are utilized in CNTs production are but are not limited to, thermal CVD [144], plasma-assisted thermal CVD [145], surface wave microwave plasma CVD, hot filament CVD, catalytic CVD [68], etc. However, amongst the various CVD methods pyrolysis accompanied by catalytic chemical vapour deposition (CVD) is one of the widely adopted for good quality and optimum yield CNTs growth methods from plastics [146].

**Fig. 3:**
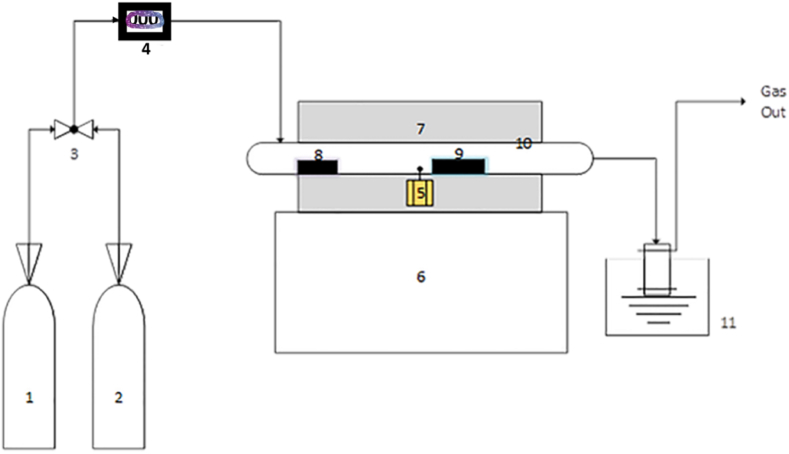
Simple form of single-stage CVD setup: 1 = H_2_/Ar (reducing) gas cylinder, 2 = Inert (N_2_) gas cylinder, 3 = Control valve, 4 = Mass flow controller, 5 = Temperature controller, 6 = Furnace holder, 7 = Electric furnace, 8 = Plastic material, 9 = Catalyst, 10 = Quartz tube reactor, 11 = Condenser system.

Apart from single-stage CNTs production methods in autoclaves [147,148], muffle furnaces [45,117], etc. Single-stage CVD reactors and two-stage CVD reactors have also been employed in CNTs growth from plastics. In the single-stage CVD technique, both stages of plastic pyrolysis and catalytic growth take place separately in one reactor using either quartz or stainless-steel tubes, but in different compartments (heating region) of the heated reactor, as shown in Fig. 3. Since plastics decomposition temperature is far lower than the synthesis (catalysis/CNTs growth) temperature. Modekwe et al. [90] employed a single-stage CVD technique in the synthesis of MWCNTs from waste PP plastics over a MgO-supported bimetallic NiMo catalyst. According to their study, a quartz tube reactor was placed into a horizontal tube furnace, where PP pyrolysis and both in-situ catalyst reduction and CNTs synthesis were carried out in a single reactor at 700 °C under N_2_ inert atmosphere. The catalyst was loaded in a separate boat and placed at the center of the reactor and was reduced in-situ before introducing waste PP in a different boat and positioned at the lower temperature region of the reactor. Gases released from waste PP degradation were deposited over the catalyst and utilized in nanomaterial growth for 30 min. Similarly, an earlier study by Mishra et al. [122] utilized single-stage CVD to investigate the use of waste PP as feedstock to synthesis MWCNTs. Both PP pyrolysis and catalytic deposition processes occurred in a single reactor where PP and catalyst were placed in two independent boats. Ni was used as a catalyst under H_2_ and Ar gas atmosphere at a synthesis temperature of 600–800 °C for 1 h.

**Fig. 4:**
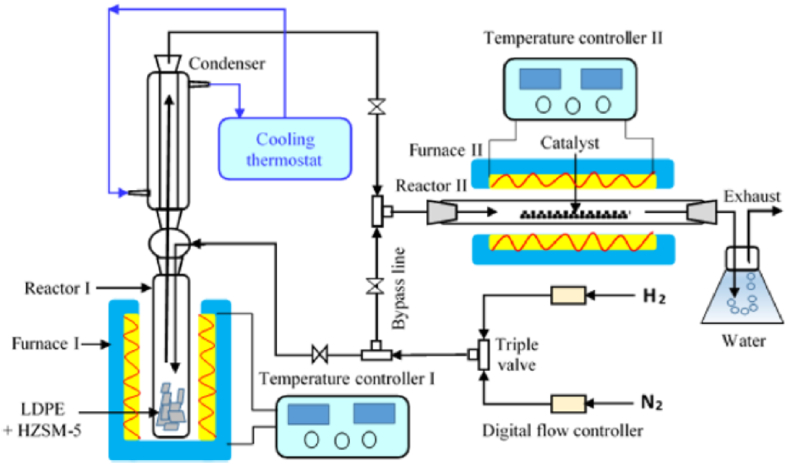
Representation of a two-stage reactor system consisting of vertical catalytic pyrolysis and horizontal catalytic growth reactors utilized in CNTs synthesis from LDPE plastic waste. Adapted from Ref. [64] with copyright permission from Elsevier BV.

In the two-stage CVD technique, the two stages of pyrolysis (or gasification) and catalytic growth are separately carried out in two individually heated and controlled reactors. This system is studied to give better control over CVD/process parameters and scaling-up for large-scale production of CNTs than other techniques and reactor designs [48], as discussed earlier. Fig. 4 depicts an example of a two-stage system consisting of a vertical and horizontal reactor system. Catalytic cracking (pyrolysis) of waste plastics (LDPE) in the presence of HZSM-5 catalyst was carried out in the vertical reactor, where a blend of condensable and non-condensable hydrocarbons was produced and channelled through the condenser to the second horizontal reactor for the catalytic growth of CNTs.

Aboul-Enein and co-workers [79] employed a two-stage pyrolysis-catalysis CVD system to investigate the production of CNTs using waste LDPE as hydrocarbon feedstock. The influence of pyrolysis and decomposition temperatures on the yield and quality of produced nanomaterials was also studied. Two independent horizontal quartz reactor systems controlled and monitored by two separate electric furnaces were utilized in the thermal pyrolysis of waste LDPE and catalytic decomposition of pyrolyzed gases. Waste LDPE was pyrolyzed in the first reactor at a temperature range of 500–800 °C and the mixture of produced gaseous hydrocarbons was channelled directly under N_2_ gas to the second reactor. In the second reactor, off-gases from the first reactor were deposited over NiMo/Al_2_O_3_ catalyst at a temperature range of 600–800 °C. Optimum CNTs yield was achieved at 700 and 650 °C pyrolysis and decomposition temperatures, respectively.

Nahil et al. [57] used a two-stage pyrolysis-catalytic steam reforming/gasification reactor system to explore the influence of metal addition on Ni-based ternary mixed oxide catalysts (Ni-Metal-Al, where M = Mn, Mg, Zn, Ce, or Ca) for the co-production of CNTs and H_2_ from waste PP plastics. In the two-stage fixed bed reactor system, waste PP was pyrolyzed at 500 °C in the first stage under N_2_ gas and pyrolysis fractions flew directly into the second stage where water (water flow rate 4.74 g/h) was contacted in the second stage, and were catalytically reformed/gasified at 800 °C. The highest CNTs yield was obtained over Ni–Mn–Al.

**Fig. 5:**
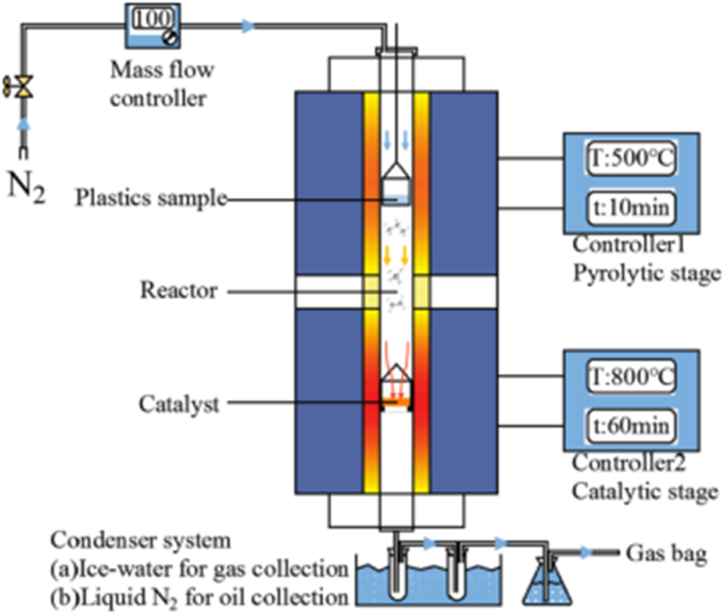
Schematic diagram of two vertical furnace units used for synthesizing CNTs by two-stage pyrolysis catalysis CVD method using different plastic types. Reproduced from Ref. [115] with permission from Elsevier.

Cai et al. [115] utilized a two-stage pyrolysis-catalysis system to investigate the link between yield and composition of the gaseous and liquid products obtained from the thermal conversion of five different waste plastic types (GPPS, PP, HDPE, HIPS, and LDPE) on the structure and quality of produced CNTs in the presence of Fe/Al_2_O_3_ catalyst. Two vertical fixed-bed quartz reactor systems were heated by two separate electrical furnaces, and each process of pyrolysis and catalysis was separately controlled at 500 °C and 800 °C respectively, as shown in Fig. 5. Each of the two processes lasted for 0.5 h under N_2_ gas atmosphere.

**Fig. 6:**
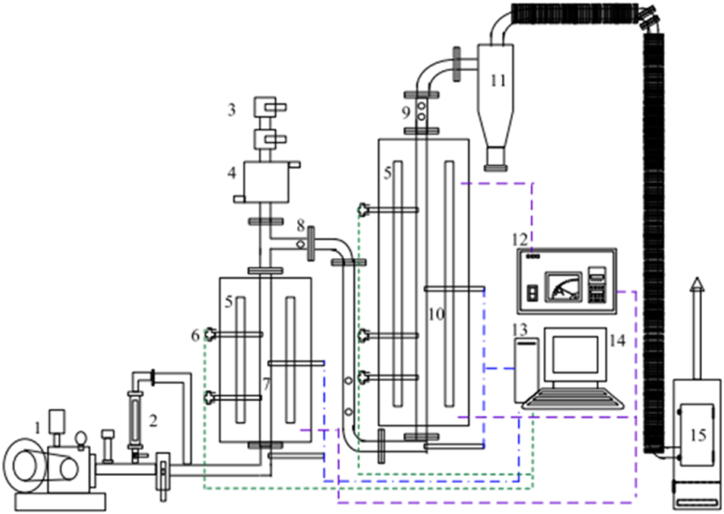
Two-stage fluidized catalytic gasification system for the production of CNTs from waste mixed plastics: (1) blower, (2) flow meter, (3) feeder, (4) cooling jacket, (5) electric resistance, (6) thermocouple, (7) first-stage bed, (8) first-stage sampling point, (9) second-stage sampling point, (10) second-stage bed, (11), cyclone, (12) TIC, (13) pressure transducer, (14) computer, (15) induced fan. Adapted from Ref. [124] with permission from Elsevier.

Yang et al. [124] used a two-stage fluidized catalytic bed to investigate the co-production of CNTs and H_2_ from the gasification of waste plastics containing a mixture of PE and PP using Ni/Al-SBA-15 and Ni–Cu/CaO–SiO_2_ as catalyst in the first and second gasifier chambers, respectively. Silica sand was utilized as the fluidized medium. The operating temperatures of the first and second chambers ranged from 600 to 800 °C, and equivalent ratios (ERs) of 0.1–0.3. CNTs were formed in the first-stage gasifier chamber as waste plastics were continuously fed into the first-chamber. They were gasified into light hydrocarbons, liquids, and solid heavy hydrocarbon fractions. Light hydrocarbons produced in the first chamber were catalyzed by Ni/Al-SBA-15 catalyst to form nanotubes. The remaining fractions were streamed into the second bed, where they were upgraded with a Ni–Cu/CaO–SiO_2_ catalyst to generate H_2_ as shown in Fig. 6. Their study concluded that the optimal operating parameters for the fluidized catalytic bed for high CNTs and H_2_ production were at ERs of 0.1 and reaction temperature for the first reactor and the second catalytic reactor were 600 °C and 800 °C, respectively.

Table 2 shows various techniques and reactors designs utilized in the synthesis of CNTs from plastic materials.

**Table 2:** Some of the reactors/techniques used in the synthesis of CNTs from plastics.

Process/Reactor Design	Polymer	Reactor/Reaction Details	Product Description	Quality/Purity of produced CNTs	Reference
Plasma catalytic decomposition using 600W, 2.5 GHz commercial microwave irradiated oven	PE	Silicone substrate-coated Fe (III) nitrate and PE was placed inside the reaction chamber and microwave irradiated to form plasma at 750 °C and 0.81 mbar.	Intertwined bundles of mixed CNTs of both SWCNTs and MWCNTs. SWCNTs diameter = 1.83 nm MWCNTs diameter = 25 nm, CNTs Length up to 0.85 μm.	Purity – 98 %	[149]
Thermal decomposition in autoclave	LDPE	The reactor with 20 % cobalt acetate and polymer was heated under N_2_ at an inside reactor pressure of about 68 atms and an operating temperature of 700 °C for 2 h.	40 % CNTs yield with an average outer and inner diameter of 100 nm and 30–40 nm respectively, and CNTs length of several micrometers.	I_D_/I_G_ - 1.1	[150]
Three-zone horizontal quartz tube reactor (decomposition-catalysis process)	Waste LDPE	Decomposition of LDPE occurred in the 1st zone of the furnace (450–550 °C), pyrolyzed gases are transferred by N_2_ to the 2nd zone where the decomposition products were preheated at 700 °C before being transferred to the 3rd zone where they interact with the catalyst; (cenospheres (SiO_2_ & Al_2_O_3_) impregnated on ferrous nitrate) in the 3rd zone where CNTs growth was initiated at 800 °C for 30 min and N_2_ flow rate of 450 cm^3^/min.	The diameter of formed CNTs was 50–60 nm		[151]
Bubbling fluidized bed reactor (Fluidized pyrolysis-catalysis bed)	PP	The polymer was fed from the top reactor (made of stainless steel) where the polymer was pyrolyzed and produced gases transferred to the fluidized bed made of quartz-rich sand or aluminium for subsequent CNT growth (N_2_ was utilized to fluidize the reactor). Bed temperature was 450–850 °C and gas fluidization velocity at 0.05–0.4 m/s	Coiled and straight nanostructures composed of MWCNTs and tubular nanofibers. Mean diameter of CNTs - 15–40 nm. Yield of produced - 85–90 wt%.		[144]
Pyrolysis-combustion-catalysis system	HDPE & LDPE (waste & virgin)	Three staged process was utilized, in the 1st stage, the sample was pyrolyzed at 800 °C under N_2_ and pyrolyzed gases were passed into a venturi in the 2nd stage where they were premixed with air or O_2_-rich air. Formed premixed flames of CO, CO_2,_ and other pyro-synthesized hydrocarbons are formed. In the 3rd stage, combusted products were deposited on 304 stainless steel (as the catalyst) at 750 °C. Other conditions: synthesis time was 30s, silicon carbide (SiC) honeycomb was used as a filter, and 50 % O_2_ mole fraction was used at the venturi.	This process produced majorly tubular MWCNTs. Length of CNTs = 1–5 μm, CNTs diameter = 15–84 nm Yield – 85 mg CNT/g polymer (about 10 % by mass).		[152]
Microwave irradiated quartz tube system	PET	Reduced Fe thin film coated Si wafer and PET were positioned in the microwave irradiated region of the reactor powered at 1000W for 2 min, under Ar flow of 500 sccm and 1.0 Torr for CNTs growth.	Vertically aligned CNT with an average diameter of 20–30 nm and a growth rate of 2.5 μm/min.	I_G_/I_D_ - 1.27	[145]

## 7 Practical applications of plastic-derived CNTs

The outstanding stiffness, strength, thermal stability, and toughness properties of CNTs [153,154] have made CNTs applicable in advanced structural materials as reinforcement materials in polymer nanocomposites [144,155]. Borsodi et al. [156] utilized CNTs produced from the gaseous products obtained from the pyrolysis of different plastics (PE, PP, PS, PA, PVC, and municipal plastics wastes) as reinforcement in the LDPE polymer matrix. Their study showed that the tensile properties of the CNT-LDPE composite were improved from 16.5 MPa for commercial virgin LDPE to 16.9–23.5 MPa for the CNTs reinforced LDPE polymer. Wu et al. [157] have also used waste PP-derived CNTs as reinforcement material in LDPE plastic. They reported that the mechanical properties of virgin LDPE were significantly improved when 2 wt% of plastic-derived CNTs were added to the LDPE polymer matrix to form the reinforced composite. The enhanced flexural and tensile strength of CNTs-LDPE composite were 8.4–9.3 MPa and 11.4–13.1 MPa, respectively, compared to 7.5 MPa flexural and 11.4 MPa tensile strength for virgin LDPE. Furthermore, the stiffness of the virgin LDPE matrix was improved from 348-495 MPa to 527.4–582.0 MPa for CNTs-LDPE composite. Likewise, the Charpy impact strength of reinforced LDPE was also improved by 10 %. A study by Yildirir et al. [158] reported the mechanical properties of composites prepared using pretreatment and melt-compounding methods by incorporating 15 % recovered non-oxidized carbon fibers as reinforcement in LDPE polymer matrix. Their result showed that the tensile strength, flexural strength, and tensile modulus of the obtained composite were 16.5, 7.5, and 571 MPa, respectively. Comparing this result (the study using recovered unmodified carbon fibres, as shown above) with the reported results on the mechanical properties of LDPE nanocomposites obtained using plastic-derived CNTs as reinforcement materials in a similar LDPE polymer matrix, it could be observed that plastic-derived CNTs exhibited satisfactory mechanical properties, and the obtained results are significantly comparable to their carbon fibres counterparts. A recent study by Wang et al. [159] uniformly dispersed waste plastic-derived CNTs as filler material in an epoxy resin matrix. The study reported that the fabricated epoxy composite reinforce with plastic-derived CNTs displayed improved mechanical properties compared to the unmodified epoxy resin.

Low-cost waste plastics have been converted into useful value-added CNTs materials, which are utilized in energy storage devices as auspicious electrode materials for storing energy in devices [160]. Employing plastics-derived CNTs as high-performance electrode materials for electrochemical supercapacitor applications is a promising sustainable approach to recycling and reusing waste. Moo et al. [161] synthesized CNTs from LDPE, PP, and mixed plastics and utilized the plastic-derived CNTs as electrode material in ORR (oxygen reduction reaction) for energy-storage materials applications. They reported improved electrochemical behaviour and performance of plastic derived-CNTs with potentials around −0.11 to −0.14 V. This high performance according to the authors was due to the high density of defective edge carbon sites present. Veksha and co-workers [162] harnessed MWCNTs obtained from the catalytic degradation of waste PET plastics (PET-12 and PET-28) as electrode materials in hydrogen evolution reaction (HER) and oxygen reduction reactions (ORR). According to their report, PET-derived MWCNTs performed better than commercially available MWCNTs and Pt-based electrode materials during the oxygen reduction reaction testing. Abbas et al. [163] employed MWCNTs derived from flexible plastic packaging waste as electrode material for application in supercapacitors. In their study, MWCNT was developed using a three-stage synthesis technique, and the as-produced nanotubes were further functionalized using nitric acid to anchor hydroxyl and carboxyl groups on the surfaces of the nanotubes. Reported results showed that synthesized CNTs from packaging wastes possess superior properties and exhibited improved electrochemical (supercapacitive) performance compared to commercial nanotubes. Similarly, few-walled CNTs derived from shoe waste plastics (composed of ethylene-vinyl acetate (EVA) and polyurethane (PU)) have been utilized and reported by Chen and co-workers [164] as electrocatalysts in carbon dioxide (CO_2_) reduction reaction. The electrocatalytic performance of the fabricated waste plastic (PU and EVA)-derived electrode materials was reported to possess 85–95 % faradaic efficiency and a CO current density of over 9 mA/cm^−2^ at low potential.

Taking advantage of the excellent electrical conductivity, structure stability, and high surface area features of CNTs, plastic-derived CNTs have also been applied as catalysts for several chemical reactions. Given the flexibility of changing their chemical composition by doping and the possibility of functionalizing the surfaces by changing their hydrophilicity [165]. Cai et al. [166] synthesized CNTs from catalytic pyrolysis of waste PP over Fe–Ni catalyst. The obtained FeNi-OCNTs composite was employed as an efficient catalyst (electrocatalysts) during an oxygen reduction reaction (ORR). The electrocatalytic performance of the synthesized FeNi-OCNT catalyst was analogous to that of the commercial 20 % Pt/C catalyst. Similarly, plastics-derived MWCNT and microspheres (mainly composed of CNTs) obtained from the thermal decomposition of PP-maleic anhydride-ferrocene composites were utilized as CNT-based catalysts in the preparation of methanol and dimethoxy-ethane during the oxidation of dimethyl ether [147]. Again, Cai and co-workers [167] also utilized iron- and nitrogen-co-doped CNTs (Fe–N-CNTs) catalysts prepared from waste plastics for use as ORR catalysts in fuel cells and metal-air-batteries.

The superior surface area and high aspect ratio features of CNTs have also been advantageous in their application as adsorbents for removing organic and inorganic pollutants from wastewater. Zheng et al. [106] utilized plastics-derived CNTs as an adsorbent in removing Methylene blue (organic dye) pollutants from aqueous media. The adsorption capacity of the synthesized plastic-derived CNTs for MB removal was 33.1 mg/g. Deokar et al. [168] also applied CNT synthesized from waste PE as an adsorbent in the removal of diuron herbicides from wastewater. Optimal diuron removal was attained using 0.4 g/L CNT dosage. Using the Hill isotherm, the authors reported that the adsorption capacity of as-produced CNTs for diuron removal was 40.37 mg/g at 30 °C. Altalhi et al. [169] also grew CNTs inside nano-porous anodic alumina membranes (NAAMs) using LLDPE in a solvent-free CVD technique. The transport performance of the obtained CNTs-NAAMs was tested using several dye molecules with positive, neutral, and negative charges. The characterization of the CNTs-NAAMs composite showed that smooth surfaces and open-ended CNTs were grown inside the NAAMs, making them efficient for separation and filtration applications. The transport performance of fabricated CNTs-NAAMs was tested in separating two mixtures of dyes and showed to be efficient after several separation processes.

The high thermal, electrical, and optical properties of CNTs make them suitable materials in sensor technology. Zhao and co-workers [170] synthesized CNTs from plastic packaging waste, and the developed plastic-derived CNTs were used as electron carriers to detect the concentration of organic contaminants (that is, para-cresol) in laboratory wastewater. Plastic waste-derived CNTs were first immobilized with laccase, and then the biosensor was fabricated with screen-printed carbon electrodes adapted to the laccase-immobilized plastic waste-derived CNTs. Reported results showed that the detection limit of the fabricated biosensor was 0.05 ppm with a linear range of 0.2–25 ppm. Also, the on-site detection and quantification of p-cresol in laboratory wastewater was validated and consistent with the result obtained by high-performance liquid chromatography (HPLC). The composite was further applied to monitor the near-real-time Fenton degradation of p-cresol in wastewater. Recently, Ribeiro et al. [171] used plastic-derived CNTs to fabricate poly(vinylidene) PVDF polymeric composite membranes. Fabricated plastic-derived composite membranes were applied in the removal of organic micropollutant (venlafaxine) from surface water.

CNTs are used as promising nanocarriers for drug delivery to transport drugs, genes, proteins, etc. to target cells due to their exceptional characteristics, such as their outstanding chemical resistance, good thermal and well-defined surface properties, as well as the nanosized, hollow monolithic structure, and the ability to easily attach desired functional groups on their outer layers [172]. Mezni et al. [173] developed highly biocompatible carbon nanocapsules for drug delivery applications using free-standing CNTs prepared using recycled plastic bags as carbon sources. In their study, the synthesized plastic-derived nanotubes were externally functionalized with chitosan and then loaded with doxorubicin (an antineoplastic drug). Obtained chitosan-coated CNTs (Ch-CNCs) nanocomposites were further tested for their localized and slow nanocarrier property within the cellular vicinity of MDA-MB-231 TXSA (human breast cancer cell line). Reported results showed that cells treated with synthesized Ch-CNCs nanocomposites had a 500-fold enhanced death rate compared to untreated cells.

## 8 Summary and concluding remark

The synthesis of CNTs from polymers as hydrocarbon feedstocks offers an extensive and practical approach to viable environmental sustainability and circularity. The sustainable development goal calls for a better circular economy and greener synthesis methods in achieving zero waste and the “waste to wealth” goal. From an economic and environmental point of view, the utilization of plastic wastes (including both recycled plastic wastes) offers a promising alternative to the use of already scarce petroleum-derived industrial chemicals like methane, acetylene, alcohol, methanol, ethylene, cyclohexane, etc.

This review has explored the breadth of catalysts, catalyst supports, operational parameters, catalyst preparation methods utilized in the synthesis of CNTs from plastics, the reactor designs, and production techniques, in addition to the already investigated applications of such produced nanotubes were also explored here. Numerous noble metal catalysts and their alloy with other metal co-catalysts (non-transition metals) have been utilized, and their respective catalytic roles explicitly investigated. However, it is not enough to only tailor the catalyst material composition; it is also important to prepare the catalyst properly in other to provide the required nanoparticle grain size, morphology, and stability with better catalyst life such that catalyst deactivation can be suppressed. The interaction between catalyst and support is seen to be important and differs from one catalyst material and support to another. The metal-support interaction (MSI) determines the morphology and size of the catalyst, the degree of dispersion and mobility of the active metal phase, and also, the diameter, quality, and yield of the obtained CNTs. It is, therefore, necessary that more comparison studies be further explored in the area of different supporting materials with a particular carbon feedstock (type of plastics feedstock used) and metal catalyst for a holistic understanding of their growth process and the influence on yield and quality of produced CNTs.

Significant improvements have been made in utilizing various real-world plastic waste materials (both mixed and single) as cheap carbon precursors for CNTs production. Despite the colossal progress already achieved in the valorization of plastic polymers into value-added CNTs. Little work has been considered in some parameters such as gas environment and their flow rates, since different vacuum environments and special ambient gases are necessary in preventing high-temperature oxidation of carbon. More practical applications of plastic-derived CNTs nevertheless need to be explored especially in purity-sensitive and structure-alignment applications.

Despite the huge efforts recorded in the catalyst design and engineering, significant improvements are still necessary for the design of catalysts from waste materials in other to minimize the need for commercially sourced materials and enhance the economies of the processing steps needed to create the desired catalyst nanoparticle and size. The economic and environmental benefits of plastics waste-derived CNTs could be negated if their synthesis utilizes huge energy-intensive procedures, expensive treatment or purification steps, or releases considerable quantities of harmful or toxic gases and pollution. However, despite these issues, the principal idea of seeking value from waste streams aligns perfectly with the pursuit of a sustainable environment and circularity and will become increasingly important in the modern world, facing the burgeoning challenges of land, air, and water pollution arising from plastic solid wastes.

## Ethics approval and consent to participate

Not applicable.

## Consent for publication

Not applicable.

## Funding statement

This work was supported by the 10.13039/501100006565University of Johannesburg (10.13039/501100006565UJ), South Africa, under the Global Excellence Stature (GES) Fellowship 4.0.

## Data availability statement

Data will be available on request.

## Additional information

No additional information is available for this paper.

## CRediT authorship contribution statement

**Helen U. Modekwe:** Writing – review & editing, Writing – original draft, Funding acquisition, Conceptualization. **Michael O. Daramola:** Writing – review & editing, Supervision, Conceptualization. **Messai A. Mamo:** Writing – review & editing, Supervision, Conceptualization. **Kapil Moothi:** Writing – review & editing, Supervision, Conceptualization.

## Declaration of competing interest

The authors declare that they have no known competing financial interests or personal relationships that could have appeared to influence the work reported in this paper.
